# Mitochondria and Mitochondrial DNA: Key Elements in the Pathogenesis and Exacerbation of the Inflammatory State Caused by COVID-19

**DOI:** 10.3390/medicina57090928

**Published:** 2021-09-03

**Authors:** José J. Valdés-Aguayo, Idalia Garza-Veloz, José I. Badillo-Almaráz, Sofia Bernal-Silva, Maria C. Martínez-Vázquez, Vladimir Juárez-Alcalá, José R. Vargas-Rodríguez, María L. Gaeta-Velasco, Carolina González-Fuentes, Lorena Ávila-Carrasco, Margarita L. Martinez-Fierro

**Affiliations:** 1 Molecular Medicine Laboratory, Unidad Académica de Medicina Humana y C.S., Universidad Autónoma de Zacatecas, Carretera Zacatecas-Guadalajara Km.6. Ejido la Escondida, Zacatecas 98160, Mexico; josejuan104@gmail.com (J.J.V.-A.); idaliagv@uaz.edu.mx (I.G.-V.); jibadillo@hotmail.com (J.I.B.-A.); calibgst@gmail.com (M.C.M.-V.); vladimir.j.a@uaz.edu.mx (V.J.-A.); jrvr159@gmail.com (J.R.V.-R.); doctoralac@gmail.com (L.Á.-C.); 2 Microbiology Department, Facultad de Medicina, Universidad Autónoma de San Luis Potosí, Avenida Venustiano Carranza 2405, San Luis Potosí 78210, Mexico; sofia.bernal@uaslp.mx; 3 Hospital General de Zacatecas “Luz González Cosío”, Circuito Ciudad Gobierno 410, Col. Ciudad Gobierno, Zacatecas 98160, Mexico; lucitagaeta@hotmail.com (M.L.G.-V.); carolina.glz.fuentes@gmail.com (C.G.-F.)

**Keywords:** mitochondrial DNA, mitochondrial dysfunction, COVID-19, SARS-CoV-2

## Abstract

*Background and Objectives*. The importance of mitochondria in inflammatory pathologies, besides providing energy, is associated with the release of mitochondrial damage products, such as mitochondrial DNA (mt-DNA), which may perpetuate inflammation. In this review, we aimed to show the importance of mitochondria, as organelles that produce energy and intervene in multiple pathologies, focusing mainly in COVID-19 and using multiple molecular mechanisms that allow for the replication and maintenance of the viral genome, leading to the exacerbation and spread of the inflammatory response. The evidence suggests that mitochondria are implicated in the replication of severe acute respiratory syndrome coronavirus 2 (SARS-CoV-2), which forms double-membrane vesicles and evades detection by the cell defense system. These mitochondrion-hijacking vesicles damage the integrity of the mitochondrion’s membrane, releasing mt-DNA into circulation and triggering the activation of innate immunity, which may contribute to an exacerbation of the pro-inflammatory state. *Conclusions*. While mitochondrial dysfunction in COVID-19 continues to be studied, the use of mt-DNA as an indicator of prognosis and severity is a potential area yet to be explored.

## 1. Introduction

Coronavirus disease 19, (COVID-19) is a disease caused by the infection of the severe acute respiratory syndrome coronavirus 2 (SARS-CoV-2), which was identified for the first time in the city of Wuhan, in the Hubei Province, China in December 2019, causing a global epidemic that has presented a significant threat to the health of the world’s population. As of September 2021, there have been approximately 218,921,481 confirmed cases of COVID-19 around the world and approximately 4,549,975 deaths [1].

SARS-CoV-2 belongs to the beta subgroup of coronaviruses. This virus measures approximately 120–160 nm in diameter, with a genome made up of a positively charged RNA [2] and a petal-shaped projection, called the spike (S) protein. This S protein mediates virus binding and membrane fusion during the infection [3].

SARS-CoV-2 enters host cells by the mechanism of viral spike protein binding to the surface receptor for angiotensin-converting enzyme 2 (ACE-2), which mostly allows for the entry of the virus into type II pneumocytes in the lung of the host [4]. This induces a local inflammatory process and promotes the release of multiple cytokines, such as tumor necrosis factor alpha (TNF-a) or interleukin-1 beta (IL-1B) and IL-6, which recruit circulatory leukocytes and amplify the systemic inflammation [5].

**Figure 1:**
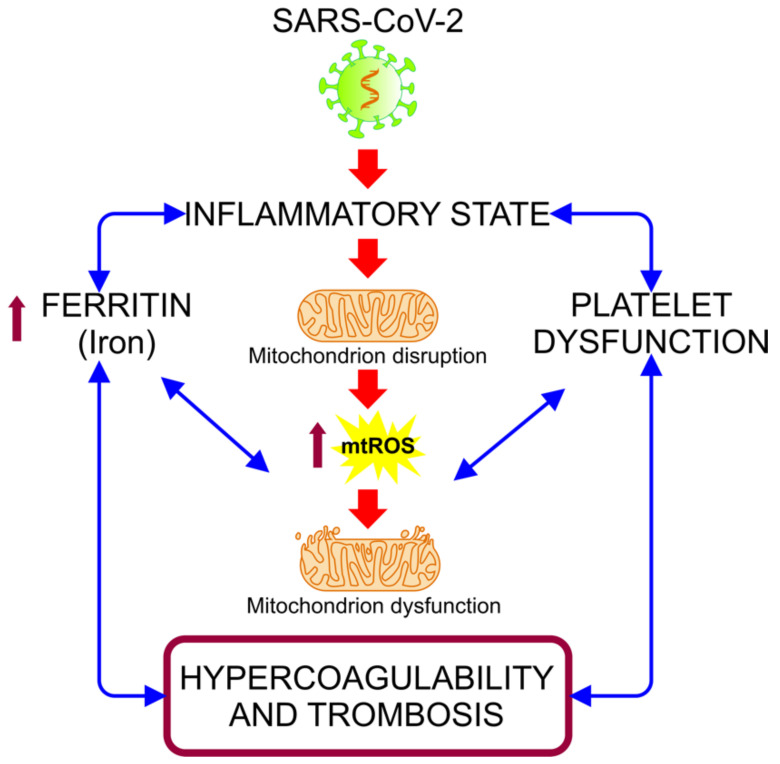
Alteration of iron homeostasis and states of hypercoagulability and thrombosis caused by the hyper-inflammatory state associated with COVID-19. The relationship between the inflammation caused by SARS-CoV-2 infection and the increased ferritin and platelet dysfunction generates a vicious cycle, in which the mitochondrial dysfunction intensifies, inducing an increase of the mitochondrial reactive oxygen species (mt-ROS) and causing an exacerbation of this cycle, resulting in a state of hypercoagulability and thrombosis [9].

The biochemical and pathological features produced by COVID-19 leads to a state of acute inflammation, which is correlated with subsequent destructive effects, manifested as persisting hypoxia, hypercoagulability, acidosis, and altered aerobic glycolytic metabolism, with an elevated lactate dehydrogenase (LDH) and subsequently lactic acidosis [6]. This inflammatory condition could be related to hypermetabolic states, such as hyperglycemia, and with cellular alterations, such as mitochondrial dysfunction, due to its various functions in metabolic pathways and cellular functions [7,8]. The hyper-inflammatory state associated with COVID-19 induces oxidative stress (OS) events, alterations in iron homeostasis, and states of hypercoagulability and thrombosis (Figure 1). These events are closely related to mitochondria, organelles that have received minor attention in connection with the pathogenesis of COVID-19, despite being essential in organs and cells with a high metabolism that are highly affected in this disease, such as hepatocytes, pneumocytes, endothelial cells, cardiomyocytes, renal cells, and neurons [7].

In this review, we aimed to show the importance of mitochondria, as organelles that, in addition to producing energy necessary for the survival of eukaryotic cells, intervene in the pathological process of multiple pathologies, including COVID-19. This intervention occurs through multiple unfamiliar molecular mechanisms that allow for the entry, replication, and maintenance of the viral genome, causing mitochondrial dysfunction and membrane damage and releasing mitochondrial DNA into circulation, which spread and exacerbate the inflammatory response. Understanding these mechanisms could guide us in the use of mitochondrial damage products, such as mitochondrial DNA (mt-DNA), as a prognostic and severity factor in patients requiring hospitalization for COVID-19.

## 2. General Concepts

### 2.1. Infection and Replication of SARS-CoV-2

The most important route of transmission of SARS-CoV-2 is from Flugge droplets; however, in susceptible individuals, transmission can occur through other body fluids with viral content, such as sputum, saliva, urine, and even feces, whose particles mainly pass through the mucous membranes of the oral and nasal cavities [10]. Patients with severe COVID-19 can spread a higher amount of virus during specific medical interventions, such as assisted mechanical ventilation, non-invasive ventilation, and intubation, as a result of the generation of aerosols [11].

**Figure 2:**
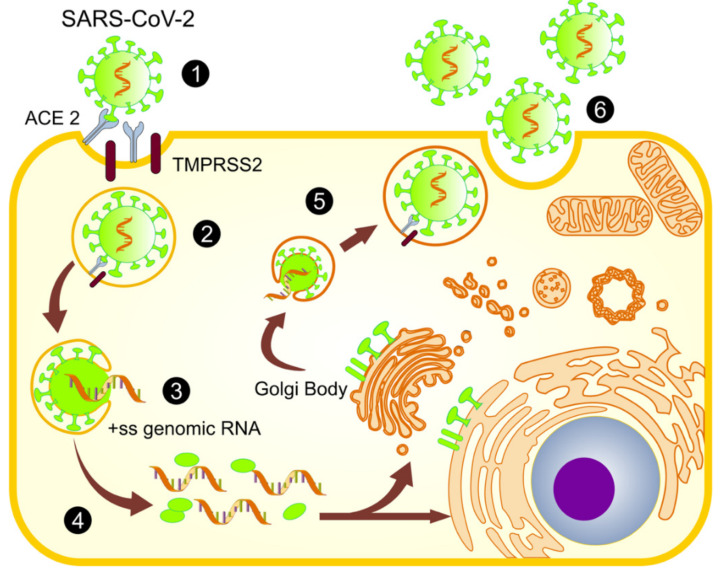
SARS-CoV-2 viral life cycle. (1) The spike protein of SARS-CoV-2 binds to receptor angiotensin-converting enzyme 2 (ACE-2), assisted by the transmembrane protease, TMPRSS2. (2) This binding promotes viral uptake and fusion at the cellular or endosomal membrane. (3) The virion releases its positive-sense single-stranded RNA (+ss RNA). (4) The release and uncoating of the incoming genomic RNA subject it to the immediate translation of two open reading frames (ORF 1 and ORF 1b), which results in the release of non-structural proteins, such as nsps, which creates a protective microenvironment for viral genomic RNA replication and transcription. (5) The translated structural proteins and newly produced genomic RNA translocate into rough endoplasmic reticulum (ER) membranes and transit through the ER-to-Golgi intermediate compartment, where they are assembled into a new virion. (6) Finally, virions are secreted from the infected cell by exocytosis [13,25,26].

While positively charged RNA viruses have their own genetic material, they only encode a limited number of proteins, requiring the host’s cellular machinery for the viral replication and assembly of viral particles [12]. The steps of the SARS-CoV-2 viral life cycle include entry into the cell, translation, replication, assembly, and exit from the infected cell (Figure 2) [13]. This dynamic process includes viral proteins in an appropriate subcellular compartment for an appropriate viral production, and this viral strategy modifies the host response to establish and perpetuate the infection. Thus, viruses develop strategies to proliferate by evading the host’s immune system, choosing specific cell organelles, such as mitochondria, nuclei, the endoplasmic reticulum (ER), and even peroxisomes, and playing an important role in the innate immunity and defense mechanisms of the host [14]. Furthermore, recent evidence indicates that viruses mainly belonging to the Flaviviridae family, such as dengue, Zika, and hepatitis C, may manipulate the mitochondria to maintain their life cycle in a similar way to SARS-CoV-2 [15].

The pathogenesis of SARS-CoV-2 begins with the cytopathic viral infection of the cellular and alveolar epithelium of the airways through its biding to the functional receptor, ACE-2 [16]. The SARS-CoV-2 S protein plays a critical role in the transmission and ongoing infection of this disease. This protein includes two main domains: the S1 domain at the N-terminal of the protein, which mediates binding with ACE-2, and the S2 domain, located at the C-terminal, which advocates the fusion of the viral membrane with the host’s cell membrane [17]. Contributing to the infectivity of SARS-CoV-2 are the serine transmembrane proteases, TMPRSS1 and TMPRSS2, which catalyze the anchoring of the S1 and S2 domains of protein S to the R677 and R797 binding sites, respectively, thus intervening in cell binding and entry [18]. ACE-2 is highly expressed in the cells of the pulmonary alveolar epithelium and in small cells of the intestinal epithelium, which are the main targets for the potential viral transmission of SARS-CoV-2 [19]. In addition, the virus is expressed in a smaller quantity in vascular endothelial cells [20], smooth muscle cells [21], proximal tubular epithelium cells [22], parietal epithelium cells [19], and kidney podocytes [23], and it has even been found in neuronal cells of the central nervous system (CNS) [24]. All these cells are characterized by a high metabolism and mitochondrial volume, along with being the most affected cells of the inflammatory and fibrotic processes typical of COVID-19.

### 2.2. Structure and Dynamics of Mitochondria

Mitochondria are described as organelles made up of two functional membranes: the outer membrane (OMM) and the inner membrane (IMM), that confine the mitochondrial matrix (MM). The OMM contains multiple proteins, including the mitochondrial voltage-dependent anion channel (VDAC) (also named mitochondrial porin), antiapoptotic proteins, such as B-cell lymphoma 2 (Bcl-2), and mitochondrial antiviral signaling protein (MAVS), which are involved in the recognition of the virus [27]. The IMM is impermeable to most small molecules and ions and contains the electron transport chain (ETC) and other membrane transporters that intervene in the ATP production. The IMM enclose the MM, that consists mainly of mt-DNA, nucleotides, and soluble enzymes, among other elements [28]. Mitochondria contain their own circular genome, mt-DNA, which is replicated independently of the host genome and has been reduced during evolution through the transfer of genes to the nucleus [29].

Mitochondria are actively in contact with multiple regulation systems and structures that allow a proper cell metabolism. An example of this interrelation is the intimate communication between ER and the mitochondria [30]. This close connection consists of mitochondrial reticular and branched networks, called mitochondria-associated membranes (MAMs), located in the cytosol, which interact dynamically and intimately with the ER network [31]. The ER lumen is considered a major intracellular Ca^2+^ storage compartment, and the depletion of the ER Ca^2+^ content is followed by its accumulation inside the MM through the mitochondrial calcium uniporter (MCU) complex [32]. The Ca^2+^ released from the ER is transferred to the mitochondrial intermembrane space by mitochondrial porins, known as VDAC [33]. An impaired Ca^2+^ handling leads to a MM Ca^2+^ overload, which has been related to important alterations in mitochondrial functions, such as an increased generation of reactive oxygen species (ROS), decreased ATP production, and cell death [34,35].

Mitochondria can change their position and morphology in cells through organized cycles of fusion (the union of two mitochondria, resulting in one mitochondrion) and fission (the division of one mitochondrion into two daughter mitochondria) to regulate their own functions and cell metabolism [36]. In recent years, these mitochondrial shape changes, called “mitochondrial dynamics”, have gained attention because of their importance in ensuring the segregation of mt-DNA, calcium homeostasis, oxidative phosphorylation (OXPHOS) modulation, and the regulation of mt-ROS levels [37].

#### Mitochondrial DNA (mt-DNA)

Mitochondrial DNA is a double-stranded circular molecule of 16,569 base pairs, with a molecular mass of 1.0 × 10^7^ daltons [38]. It is mainly associated with nucleoids and proteins located among the MM in close proximity to the mitochondrial membrane [39]. Mt-DNA encodes only 13 polypeptides that are components of the OXPHOS complexes located in the IMM [29]. The mitochondrial genome also encodes for 22 transfer RNAs (tRNA) and 2 ribosomal RNAs (rRNA), which participate in the mitochondrial translation [40]. Accordingly, most of the mitochondrial proteins are encoded by the nuclear genome and are imported to the mitochondria by translocation systems localized on the mitochondrial OMM and IMM [41].

The maintenance of the integrity of mt-DNA is complicated due to the mt-DNA vulnerability to damage, especially through the generation of oxidative damage. There are potential reasons to explain the increased sensitivity of mt-DNA to oxidative lesions [42]. First, unlike genomic DNA, mt-DNA lack histones [29]. Second, mt-DNA reside near the site of mt-ROS production in the mitochondrial membrane. Further, mt-DNA have been shown to be more vulnerable to damage as consequence of aging, potentially due to the increase of ROS production and the decreased mt-DNA repair capacity [43]. Similarly, mt-DNA mutations and deletions are implicated in many severe diseases, including mitochondrial encephalopathy, lactic acidosis, Pearson syndrome, obesity, and glucose intolerance [44,45].

### 2.3. Mitochondrial Functions

Mitochondria are considered essential for eukaryotic life due to their participation in several metabolic and intracellular signaling networks that regulate various cellular functions, including ATP synthesis, ROS regulation, inflammation, lipid and iron metabolism, apoptosis, and even the regulation of the synthesis of some hormones, such as cortisol or estrogen [46].

#### 2.3.1. ATP Production

Mitochondria are responsible of multiple reactions that result in a free-energy production by the adenosine triphosphate (ATP) formation, which is the effective central link between energy-demanding and energy-producing processes [47]. The main energy source of cell metabolism is glucose, which is catabolized in three different processes: glycolysis, tricarboxylic acid cycle (TCA), and oxidative phosphorylation (OXPHOS) in order to create ATP [48]. Glycolysis is a process in which glucose is converted through a series of catalyzed reactions into two pyruvate molecules, which enter into the TCA and are oxidized to acetyl coenzyme A (Acetyl-CoA) and CO_2_. The energy released in this oxidation, provide energy for the respiratory chain in the form of electrons [49], which are donated to the ETC, located in the IMM. The ETC consists of four protein machines or complexes (I, II, III, and IV), which, through consecutive redox reactions, withstand conformational changes to pump protons from the matrix to the intermembrane space (IMS) [50] to generate an electrical potential used by the ATP synthase to catalyze the ATP phosphorylation and the subsequent ATP production.

Furthermore, mitochondria are an elemental part of the lipid biosynthetic pathways, including the fatty acid beta-oxidation pathway, whose function is to convert long-chain fatty acids into acyl coenzyme A (Acyl-CoA). In the MM, Acyl-CoA is oxidized into Acetyl-CoA, fueling the TCA and creating ATP, making fats an essential element in biochemical energy development [51].

#### 2.3.2. Mitochondria and the Production of Reactive Oxygen Species (ROS)

Mitochondria are an important source of cellular ROS, as they have eleven identified sites for the production of superoxide (O_2_^•−^) and hydrogen peroxide (H_2_O_2_) linked to electron transport and OXPHOS [52], and identified as progenitors of ROS [53]. ROS are produced during oxidative metabolism through the one-electron reduction by molecular oxygen (O_2_), forming superoxide anions (O2^•−^). Superoxide is converted in H_2_O_2_ due to the superoxide dismutase (SOD) located in the mitochondria and cytosol [54]. Complexes I, II, and III of the electron transport chain also contain sites where electrons can prematurely reduce oxygen to produce superoxide [55]. ROS are considered toxic products of aerobic metabolism and the primary cause of cellular damage; however, when produced in a controlled manner, they play important signaling roles [54].

#### 2.3.3. Mitochondria and Cell Death

Mitochondria are also involved in cell death [56]. In addition to necrosis and apoptosis, mitochondria participate in another inflammatory cell death mechanism dependent on inflammasome activation, called “pyroptosis” [57]. The inflammasome is a cytosolic protein complex that can sense pathogen-associated molecular patterns (PAMPs) or danger-associated molecular patterns (DAMPs). The mt-DNA released in the cytosol act as a DAMP that induces inflammasome oligomerization and activation in T cells and macrophages, leading to the cleavage of pro-IL-1B and pro-IL-18 to their active forms, which, in turn, leads to pyroptosis, as a pro-inflammatory cell death mechanism [58]. This process, known as the “danger model”, corresponds to the presentation of an antigen in the context of a danger signal triggering an efficient immune response, not only for the exogenous antigens, but also for the signal released by damaged or stressed tissues [59]. Therefore, inflammasomes, such as NLRP 3, are essential for the development of acute respiratory distress syndrome (ARDS) in COVID-19 and other lung diseases [60,61,62].

All these observations concerning mitochondria highlight that the events occurring inside and outside these organelles regulate the activity and organization of mitochondrial structure, with consequences for both their own behavior and the general cell environment.

### 2.4. Mitochondria and Immune Response

The immune system is a family of heterogeneous cells; essentially, it is a compound of monocytic cells (e.g., T lymphocytes, B lymphocytes, and macrophages), granulocytic cells (e.g., neutrophils, macrophages, and basophils), and cells located in peripheral regions, whose function is to present antigens to the effector cells (e.g., dendritic cells). All of these cells play diverse roles during inflammation and homeostasis in a tissue-specific manner [63]. Recent studies have demonstrated that immune cells use distinct metabolic programs to perform their functions [64]. These energy-gained mechanisms are primarily regulated by the mitochondrion, elucidating that they are involved in the differentiation and activation of immune cell processes [65].

#### 2.4.1. Neutrophils

Neutrophils are cells that belong to the innate immune system and are quick- responders during the inflammatory response. Their functions are to sense pathogens and rapidly respond to stressful situations [66]. Once at the site of inflammation, neutrophils recognize, engulf, and kill microorganisms through multiple mechanisms, such as oxidant production, degranulation, or the release of neutrophil extracellular traps (NETs) [67]. It has been recognized that mitochondria and the release of mt-DNA from the neutrophils play important roles in other neutrophils functions, such as NET release, phagocytosis, degranulation, and chemotaxis [68].

#### 2.4.2. T lymphocytes

T lymphocytes originate from bone marrow-derived hematopoietic stem cells and migrate to the thymus for maturation and subsequently selection. When they are mature, T lymphocytes migrate to the periphery. These native T lymphocytes can be CD4+ (helper T cells, Th1, Th2, and Th17) or CD8+ (cytotoxic T cells) [65]. The antigen-presenting cell (APC) process peptides migrate to the lymph nodes, where they encounter naïve T lymphocytes. This interaction promotes T cell activation and the activation of the metabolic mechanisms of mitochondria, mainly glycolysis and OXPHOS, to promote the survival of naïve T cells and their proliferation [69]. During T cell activation, low physiological levels of ROS are generated, and a H_2_O_2_ mediated oxidative signal is activated, promoting the activation of the ROS-dependent nuclear factor kappa-light-chain enhancer of the B cell (NF-kB), which is essential for T cell activation. All these important mechanisms are effectuated due to mitochondrial dynamics, which allow for an increase in mitochondrial mass when T cells are activated [70].

#### 2.4.3. B Lymphocytes

Besides being derived from lymphoid-committed precursors in the bone marrow, B cells coordinate the activation of memory cells due to their ability to produce and secrete antigen-specific antibodies after differentiating into plasma cells [71]. Additionally, B cells can act as antigen-presenting cells (APCs) that respond to infectious pathogens earlier than T lymphocytes, principally due to the presence of functional pathogen recognition receptor (PRRs), which recognize specific pathogen-associated molecular patterns (PAMPs). PRRs are classified into four groups: toll-like receptors (TLRs), nod-like-receptors (NDRs), retinoic acid-inducible gene-I (RIG-I)-like receptors (RLRs), and C-type lectin receptors (CLRs) [72]. B cell activation mechanisms during the inflammatory state consist of T cell-independent or -dependent mechanisms that include the participation of pleiotropic cytokines, such as IL-1, IL-6, IL-9, IL-17, IL-27, and TNF-a, and the recognition of PAMPs via PRRs.

Due to their high proliferative rate and antibody production, B cells need high metabolic rates, which require different glucose uptake rates and oxidant production, as a reflection of their increased number of mitochondria and anatomy changes (from an elongated to a rounded shape). Furthermore, human B cells are capable of releasing mt-DNA into the extracellular environment after the activation of TLR9 by unmethylated cytosine-phosphate-guanine (CpG) dinucleotides, which are principally responsible for activating anti-viral responses through the induction of type 1 IFN production [73].

#### 2.4.4. Mitochondria and the Activation of the Immune System

**Figure 3:**
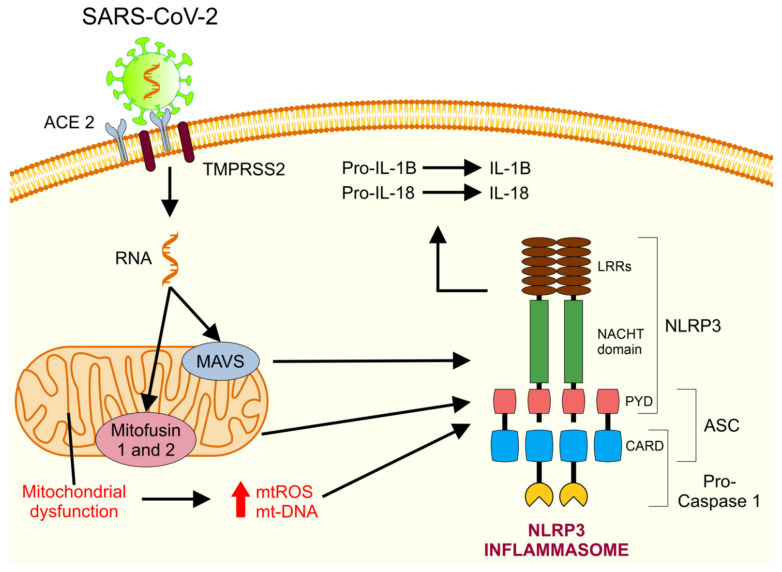
NLRP3 inflammasome activation by mitochondria. Once inside the cell, SARS-CoV-2 RNA is detected by the mitochondrial molecules, MAVS and mitofusin 1 and 2, causing mitochondrial dysfunction and the subsequent liberation of ROS and mt-DNA into the cytosol. This causes the activation and recruitment of NLRP3, ASC protein, and Caspase-1, which assemble to create the NLRP3 inflammasome. Once activated, the NLRP3 inflammasome cleaves the cytokines Pro-IL-1B and Pro-IL-18 into their mature and biologically active forms (IL-1B and IL-18), thus exacerbating the inflammation state [78]. ASC: apoptosis-associated speck-like protein containing a CARD. CARD: caspase recruitment domain. LRR: leucine-rich repeats. PYD: pyrin domain.

In addition to the impact of mitochondrial membranes on metabolism and the role of mitochondrial dynamics in the activation of innate immunity, studies highlight the importance of mitochondrial membranes as an assembly and signaling platform [74]. MAVS, located on the OMM, induces downstream antiviral signaling by promoting the release of type 1 IFN (important for clearing the virus) and activates NF-kB, which is important for triggering innate and adaptative immunities. Mitochondria also serve as a crucial signaling platform for the NLRP3 inflammasome (Figure 3) by altering mt-ROS, lipids and membrane potential [72,75]. Further, the mt-DNA released into the cytoplasm and out into the extracellular fluid act as DAMPs [76] and activate different recognition receptors and innate immune responses, including the cyclic GMP-AMP synthase pathway (cGAS-STING), toll-like receptor 9 (TLR9), and inflammasome formation, leading to an up-regulation of various type I IFN, pro-inflammatory chemokines, and cytokines [77].

These findings summarize how the mitochondrial metabolism and dynamics are linked to the shaping of immune cell function and effects. However, it is becoming clearer that mitochondria must be perceived as a functional organelle network, rather than an autonomous entity. Such networks are likely to be beneficial when immune cells are required to quickly adapt to new bioenergetic states across multiple organelle compartments. Nevertheless, this mitochondrial network system could become dangerous once one of the compartments fails and cannot be repaired, causing the spread of the defects throughout other organelles and leading to systemic cell failure [79].

## 3. Mitochondrial Dysfunction in Viral Infections

While information is still limited, there is evidence of mitochondrial alterations due to the replication and infection of multiple viruses, mainly those belonging to the Flaviviridae family [15].

Members of the Flaviviridae family replicate in the ER, using its membrane to form double-membrane vesicles for viral genome replication. ER disruptions induce an increase in Ca^2+^ excretion, causing MAMs to perform a deficient Ca^2+^ exchange between ER and mitochondria. This critical process for the mitochondrial-dependent induction of cell death promotes an excessive production of mt-ROS [80]. Studies of the Zika virus, a member of the Flaviviridae family, suggest the virus releases genetic material into the cytoplasm and binds to ER membranes, initiating the synthesis of viral proteins inside ER invaginations. This ER function alteration indicates an early stage of cell death preceding the accumulation of ROS, calcium imbalance, and mitochondrial dysfunction [15]. In comparison, studies of hepatitis C virus (HCV) relate to the union of a viral complex (NS3/NS4A) of the HCV to the OM protein, MAVS, which is an essential component of the innate immune response pathway. The viral dsRNA is recognized by a cytosolic PRR, called RIG-I, which leads to the expression of type 1 IFN via the activation of interferon regulatory factor 3 (IRF3) [81]. HCV replication induces ER stress, leading to a Ca^2+^ release from the ER into the cytoplasm. The interaction between the virus and VDAC, a major component of the mitochondrial permeability transition (MPT) pore, sensitizes the MPT pore and increases mitochondrial Ca^2+^ uniporter activity, leading to an increased Ca^2+^ influx in the mitochondria and, subsequently, an increase in the mt-ROS production. The increase of Ca^2+^ in the mitochondria leads to an alteration of the ETC by the inhibition of the electron transport Complex I, causing an electron leak from the ETC favoring H_2_O_2_ formation and increasing the mt-ROS production [81]. While the activity of Complexes II and III are not affected by HCV, HCV affects Complex IV (cytochrome C oxidase), contributing to the increase of ROS formation by the mitochondria [82]. All this activity of viral infection reflects a vicious circle: the mitochondria increase the uptake of Ca^2+^, leading to increased mt-ROS levels, which, in turn, induce MPT pore opening [83]. Besides the direct effects on the respiratory chain, the functions of the TCA and lipid beta oxidation are affected, followed by the ETC, potentially causing mitochondrial membrane alteration and contributing to the increased mt-ROS formation [84]. Table 1 shows the multiple mechanisms in which viruses, particularly from the Flaviviridae family, use mitochondria as part of their pathogenesis.

## 4. The Mitochondrial Role in SARS-CoV-2 Replication

As previously mentioned, SARS-CoV-2 uses ACE-2 to enter the cell [16]. Once in the cytoplasm, SARS-CoV-2 needs to initiate replication from a single-stranded RNA (ssRNA) through an intermediate dsRNA [12]. This process predisposes the virus to detection by TLRs and mitochondrial viral signaling systems (AVMs) [7,8,94,95,96]. SARS-CoV-2 evades this detection by inducing the production of double-membrane vesicles with the help of the mitochondria and the ER for their replication and dissemination [94]. These vesicles, in addition to being an appropriate site for replication, trick the host into not digesting them. The importance of the mitochondria for the virus replication explains the presence of the SARS-CoV-2 RNA genome and all the produced sub-genomic RNAs in the host MM and nucleolus [96,97].

**Figure 4:**
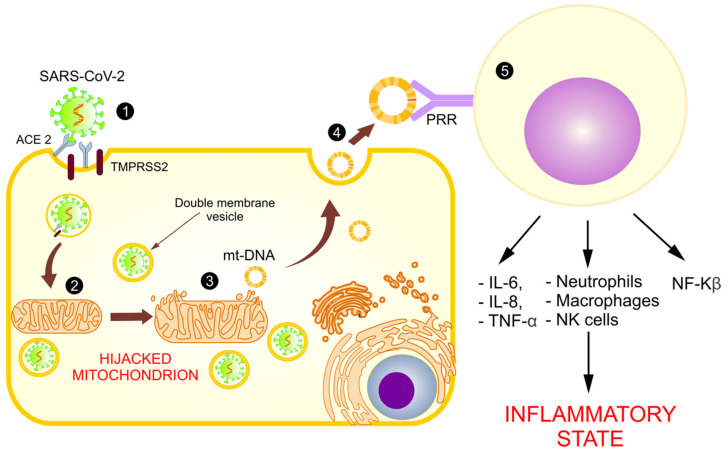
Use of mitochondria for SARS-CoV-2 replication and the release of mt-DNA into the circulation. The figure shows the introduction of SARS-CoV-2 into the host cell (1) and the hijacking of the mitochondrion for its replication (2), causing a loss of its membrane and the release of mt-DNA into the cytosol (3) and circulation (4), activating the immune system (5), and increasing the inflammatory state.

Based on these models and in the identification of an open reading frame (ORF), the SARS-CoV-2 viral genome has been identified in mitochondria, indicating that mitochondrial residency could be required for double-membrane vesicle formation, which is critical for the unstoppable replication of coronavirus, as it evades cellular defense [7]. With these findings, it is assumed that the coronavirus hijacks the mitochondria and uses their machinery for its own sustenance and replication, and the mitochondria eventually assist with its virulence and transmissibility (Figure 4). These actions may damage the mt-DNA and mitochondrial membrane, causing leakage of the altered mt-DNA into the cytoplasm, and consequently act as a trigger for innate immunity activation.

## 5. Mitochondrial Dysfunction in COVID-19

Most of the patients infected with SARS-CoV-2 showed either a mild infection, with no fever nor pneumonia, or a moderate infection, with clinical manifestations, such as cough, fever ≥ 38 °C, arthralgias, myalgias, and dyspnea [98]. Nonetheless, a severe infection caused pneumonia and respiratory failure, accompanied by other complications, such as microvascular thrombosis, coagulopathy, ARDS, disruption in iron homeostasis, and shock [99]. While the factors that interfere in the infection’s severity are not fully understood, the immune system and mitochondrial function might play an important role in the pathogenesis of the disease.

### 5.1. Mitochondria and Cytokine Storms

The hyper-inflammatory state caused by COVID-19 in the fight against SARS-CoV-2 infection is a hallmark of the release and amplification of a cytokine storm identified in patients with severe COVID-19, which is regulated by multiple mitochondrial pathways. A cytokine storm occurs due to an extensive increase in ROS, which results in the release of TNF-a, IL-1β, IL-6, and IL-18, which mediate and intensify the inflammatory state and promote the inflammasome response. As a consequence of the activation of a cytokine storm, the cells reprogram their metabolic machinery by increasing the glycolysis and reducing the Krebs cycle’s ATP production, thus inducing mitochondrial atrophy [100]. Further, inflammatory cytokines, such as TNF-a, IL-6, IL-10, and IFN-γ, are involved in the exacerbation of the inflammatory state and in mitochondrial dysfunction. TNF-a induces calcium-dependent increases in mt-ROS. Moreover, IFN-γ has been shown to upregulate genes, thus inducing mt-ROS generation and increasing intracellular OS. Finally, IL-10 and IL-6 were found to modulate mt-ROS generation by regulating the activity of the ETC [101]. These inflammatory and immune sentinels trigger intracellular cascades that alter mitochondrial metabolism and increase its dysfunction. Cytokines, such as IL-6 and TNF-a, found in the serum of patients with COVID-19, impede the mitochondrial OXPHOS pathway, causing a decrease in the ATP production and an abnormal mt-ROS production [102], which support cellular diseases and ageing. This causes mitochondrial membrane permeabilization, the release of mt-DNA, and altered mitochondrial dynamics, and it could ultimately result in cell death. It has also been demonstrated that the differential gene expression in SARS-CoV-2-infected lung cell lines shows an upregulation of the genes involved in mitochondrial inflammatory/cytokine signaling, causing a downregulation in genes involved in the mitochondrial functions of respiration, organization, and autophagy [103]. As an example, the study conducted by Zhang et al. in 2020 revealed that alveolar epithelial cells in humans with dysfunctional mitochondria show an increased production of some pro-inflammatory cytokines, such as IL-6, IL-12, chemokine ligand 8 (CXCL-8), CCL-20, CCL-3, and CCL-4, all of which were found to be increased in COVID-19 [104]. In addition, the upregulation of chemoattractants, such as CXCL-8, promotes the infiltration of neutrophils in the lung, thus contributing to ROS generation and protease activation, which inflict mitochondria damage [105].

### 5.2. COVID-19: Disruption in the Mitochondrial Regulation of Iron Homeostasis

After being recollected, the iron is stored as ferritin in the mitochondria for multiple biochemical functions, such as mt-ROS formation [106]. Ferritin is an intracellular protein, whose function is to regulate the iron deficiency or its overload via mitoferrin carriers. The disruption of cellular levels, mitochondrial iron metabolism, and mitoferrin carriers leads to hyperferritinemia, associated with hyper-inflammation and mt-DNA damage [107], which exacerbates the cellular OS and the massive release of inflammatory mediators, ROS, and free radicals. In addition, the disrupting mitochondrial homeostasis caused by the hyperferritinemia drives mitochondrial respiration from an aerobic into an anaerobic state, which favors pyruvate reduction into lactate, creating an important amount of LDH [108]. This metabolism alteration is associated with an increased conversion of NADH into NAD^+^, which inhibits the mitochondrial metabolism in an out-of-control positive feedback that intensifies the inflammation response [109].

### 5.3. Mitochondrial Disfunction in the Hypercoagulability State Associated with COVID-19 Severity

Coagulation abnormalities have been associated with the severity and aggravation of COVID-19 patients. Thrombocytopenia is reported to be more prominent in severe patients in comparison with non-severe cases [110] and is used as a prognosis factor to identify patients at risk of developing severe conditions, such as disseminated intravascular coagulation (DIC). The relation of platelets and hypercoagulable states presented in COVID-19 is associated with the absence of the genomic DNA of the platelets, causing them to preserve organelles, such as mitochondria, to maintain their structure and function. The accumulation of five-to-eight mitochondria is critical for vital platelet functions, such as metabolism, aerobic respiration, ROS generation, reduction of mitochondrial membrane potential, and platelet apoptosis [111]. Mitochondrial dysfunction in COVID-19 altered platelet survival and apoptosis, raising the mt-ROS production, which leads to severe OS, an altered mitochondrial membrane potential, and a reduced ATP production, thus increasing the risk of thrombus formation [8].

These findings contribute important evidence suggesting that mitochondrial disruption interferes with effective immune responses, increases the inflammatory state, exacerbates OS, and predisposes patients to an increased COVID-19 severity. Nevertheless, the mechanism involved in other viral infections, such as the ER associated with an altered Ca^2+^ excretion and disfunction of MAMs, has not been fully addressed in the COVID-19 pathology.

### 5.4. Role of the Mitochondrial Dysfunction in Arterial Hypertension and Diabetes: Risk Factors Associated with COVID-19

Mitochondrial dynamic and function is affected by the changes, alterations or needs in response to metabolic signals. The metabolic changes in multiple diseases, such as hypertension and diabetes, affect the form, number, and function of the mitochondria, which consequently intervene in the organ’s functionality.

The hypertension-related cardiac hypertrophy has been associated with alteration of the ETC, metabolic substrate utilization changes and ATP synthesis [112], identifying that proteins involved in mitochondrial OXPHOS were overexpressed whereas the α-subunit of the mitochondrial precursor of ATP synthesis was down-regulated [113]. These results suggest that hypertrophic hearts are related with an altered mitochondrial energetic metabolism, including decreased ATP production and cellular respiration.

Moreover, the increased ROS generation caused by reduced antioxidant capacity in cardiovascular, renal, and nervous systems, has been identified as an important role in the vascular remodeling, vasoconstrictor, and vasodilator responses and in altered vascular mechanics associated with hypertension [114]. Similarly, hyperglycemia and insulin resistance are also linked to excess ROS production [115], that subsequently trigger both mitochondrial mediated cell death and the degradation of mt-DNA [116].

Pancreas cell alteration and deficient insulin secretion play an important role in the development of insulin resistance and diabetes; further, alterations of mitochondrial dynamics have been related to disruption of pancreatic beta cells function [117]. Opa1, a GTPase associated in the mitochondrial fusion machinery, is required for an adequate function of the respiratory chain in pancreatic beta cells. Thus, the ablation of Opa1 cause mitochondrial fragmentation and death of pancreatic beta cells [118], associated with reduced insulin secretion and impaired systemic glucose homeostasis. Further, defects in glucose-stimulated mitochondrial ATP production in beta cells deficient in Opa1 may result from OXPHOS alterations, exacerbating the deficient insulin secretion.

The relation of mitochondrial functionality in the development of these diseases, associated with the severity of COVID-19, emphasizes the importance of the mitochondria in the exacerbation of this viral disease.

### 5.5. Abnormal Functioning of Mitochondria and Release of mt-DNA as DAMPs

During SARS-CoV-2 infection, hypoxia cause a switch to aerobic glycolytic respiration, which reduces the ETC Complex I–IV activity and alters the OXPHOS and mitochondrial function, inducing a condition of hyperglycemia, elevated ATP production, and increased oxygen consumption [119]. These altered metabolic pathways lead to an increase in cytoplasmic and mitochondrial ROS. The failure of damaged and senescent mitochondria to clear the ROS may damage the mitochondrial structure, biogenesis units, and lipid membranes, and finally, it may generate altered mt-DNA levels [120]. Furthermore, as the virus uses mitochondrial machinery to replicate, the integrity of the mitochondrial membrane is lost, releasing mt-DNA into the cytosol and circulation [8]. These circulating mt-DNA fragments (DAMPs), possessing functions similar to the PAMPs released by the microbes, are identified by PRRs [7]. The most well-known PRRs are TLRs, which are membrane-bound or intracellular receptors expressed by all innate immune cells with several different subtypes: TLR4 detects lipopolysaccharides (LPS), a Gram-negative bacteria membrane component; TLR2 detects cell wall components, such as peptidoglycan and lipopeptides from Gram-positive bacteria; and TLR9 detects unmethylated CpG DNA motifs of the mitochondrial genetic component [121]. The TLR9 signaling pathway results in the activation of NF-kβ and mitogen-activated protein kinases (MAPKs), which trigger a pro-inflammatory response, initially with the activation of immune cells. Activated innate immunity induces gene transcription, which leads to the production of signal molecules, such as pro-inflammatory cytokines (IL-6 and IL-8), prostaglandins, interleukins, and chemokines, and it also activates the complement system, evoking the inflammatory cascade [122].

Another function of the DAMPs is to activate the NF-Kβ pathway and inflammasomes, mainly the NLRP3 inflammasome [72]. The NLRP3 inflammasome, containing the pyrin domain-containing protein 3 (NLRP3), is activated by a large range of mitochondrial ligands, such as mt-DNA, ATP, and mt-ROS, and by inducing mitochondrial dysfunction. After the activation of NLRP3, an adaptor protein apoptosis-associated speck-like protein recruits caspase-1 to the inflammasome through a caspase recruitment domain. When caspase-1 is activated, it cleaves pro-interleukin 1B and pro-interleukin 18 to their biological forms [78], leading to an inflammation cascade. Mt-DNA, as a DAMP, could also affect immune responses by the cyclic AMP-GMP synthase (cGAS) pathway. The cGAS pathway, as part of the innate immune system, detects the presence of mt-DNA in the cytosol, triggering type 1 IFN inflammatory responses [123]. cGAS, its second messenger cyclic GMP-AMP (cGAMP) and the sensor of cGAMP, whose main function is the stimulation of interferon genes (STING), are a major sensing pathway for cytosolic DNA [124]. The stimulation of this pathway induces NF-kB activation, controlling the transcription of pro-inflammatory cytokines and chemokines [125].

Mitochondrial DAMPs are an important element in the infection detection, stimulation, and maintenance of immune response against pathogens [126]. However, the sensing of mitochondrial DAMPs may also cause an undesirable immune response and result in an unwanted inflammation. In conditions of massive cell damage, such as COVID-19 disease, protective mitochondrial mechanisms, as well as mitophagy processes in which damaged mitochondria are phagocyted, may not work or may be overwhelmed, inducing the release of mitochondrial DAMPs into the cytosol and circulation in overwhelming amounts. These actions may trigger an unwanted pro-inflammatory response [127]. These mechanisms exacerbate tissue dysfunction by involving metabolic, endocrine, cardiovascular, coagulation, immune, and thermoregulatory pathways, and they are also responsible for systemic effects and multi-organ failure [46].

From the data presented in the study of Indraneel Mittra et al., the authors refer to the possibility that exogenous nucleic acid (ENAs), such as mt-DNA, can be taken up by tissue cells. This proposal is similar to the hypothesis of “geno-methastasis”, which claims that once inside cells, ENAs have several biological actions, such as inducing genetic, cellular, and chromosomal damage. ENAs are certainly capable of activating both innate and adaptive immune systems and inducing a sterile inflammatory response [128,129]. These findings highlight the importance of the increase of circulating free mt-DNA fragments and the decrease of intact mt-DNA as a consequence of the SARS-CoV-2 infection.

## 6. mt-DNA as a Biomarker in Inflammatory Diseases

Defects in mitochondrial function have been proposed to be involved in the etiology of multiple pathologies, such as cancer and neurodegenerative and cardiovascular diseases. Since mt-DNA encodes critical proteins of the respiratory chain, it is highly possible that mt-DNA mutations could impact the respiratory function of cells and the subsequent accumulation of oxidative damage [130], thus increasing the accumulation of damaged mt-DNA. The relation between altered mt-DNA and oncogenesis malfunction explained the increased of altered mt-DNA content in cancer cells such as in prostate cancer cells [131], and other body fluids including saliva associated with head and neck cancer [132] and in serum levels of cell-free mt-DNAs in testicular cancer [133].

In a recent study, Amit Sharma et al. [134] found an increased circulating mt-DNA content in serum and cerebrospinal fluid (CSF) of Parkinson’s disease patients in comparison with healthy individuals. The increased levels of circulating mt-DNA were associated to mitochondrial dysfunction due to the substantia nigra degeneration. Similar results were obtained in the study of Jauhari et al. [135], in which they explored the role of melatonin as a neuroprotective hormone and its relation to the inhibition of cytosolic mt-DNA in accelerated neurodegenerative diseases. These results support the idea that in response to increased metabolic and OS, released mt-DNA act as a DAMP in the immune response and exacerbate the disease severity.

Interestingly, there is also evidence that mt-DNA can be decreased in inflammatory processes or degenerative diseases. In the study carried out by Hannah Lowes et al. [136] they obtained samples of lumbar cerebrospinal fluid (CSF) from 372 patients with Parkinson’s disease (PD) and 169 matched controls, obtaining a reduced level of mt-DNA in PD patients, compared to controls. The authors of the article linked this mt-DNA decrease to inflammation, body mass index (BMI), and psychosocial and physical stress. In addition, the results reported by Morandi et al. [137] identified a decrement of mt-DNA in patients with obesity in comparison with the control patients, which the authors associated with the mitochondrial dysfunction typical of this disease.

Accordingly, the different relation between the quantity of mt-DNA in degenerative and inflammatory diseases opens the opportunity for the use of mt-DNA levels as a biomarker for the disease onset and progression of COVID-19.

### mt-DNA: An Early Predictor of Severe Evolution and Mortality in Patients with COVID-19?

The knowledge of the structure and functions of mitochondria has allowed the relation between mt-DNA and SARS-CoV-2/COVID-19 to be uncovered and promoted the use of the mitochondrial genome as a biomarker for the severity and prognosis of COVID-19 patients. In a study performed by Scozzi et al. [138], it was reported that COVID-19 patients with high mt-DNA levels are more likely to require admission to the intensive care unit (ICU) and intubation, and they have a higher risk of death. In this study, 97 COVID-19-positive patients were evaluated, of which 56.7% required ICU admission, and 25.8% required invasive mechanical ventilation as a treatment for acute respiratory failure. To obtain the mt-DNA values of the patients, the quantitative PCR method was used to measure the accumulation of fragments derived from the cytochrome B gene (mt-CYTB) encoded by mitochondria within the cell-free circulating plasma mt-DNA. Plasma mt-CYTB levels were found to be elevated in the patients who died of COVID-19 and those who required intensive therapy and advanced mechanical ventilation in comparison with stable patients who survived.

Together with mitochondrion dysfunction, mt-DNA continues to be explored as a key factor that is potentially associated with mortality and morbidity in patients infected with SARS-CoV-2. Therefore, the association of mt-DNA and the severity of the disease and the interaction of mt-DNA with other organs, such as the kidneys, neurons, liver, and heart, remain important points to explore. Moreover, studies on ENAs, as mt-DNA, have received special attention because of their potential application as a non-invasive, rapid, and sensitive tool for the molecular diagnosis and monitoring of acute pathologies, as a diagnostic marker, and as a potentially powerful tool in the future [128]. However, additional studies on the use of mt-DNA in COVID-19 are necessary to identify and consider whether the increase or the decrease of circulating mt-DNA quantification intervenes in the prognosis and severity of COVID-19.

Is well accepted that several compounds that modulate mitochondrial function may to bind to and modulate receptors, transcription factors and kinases [139]. Altered activity of the transcriptional peroxisome-proliferator-activated receptor γ co-activator-1α (PGC-1α/PPARGC1A) and the hyperactivation of the mammalian target of rapamycin (mTOR) kinase are emerging as molecular underlying causes of mitochondrial alterations [139,140]. It is, therefore, likely that either stimulating the PGC-1α activity or inhibiting mTOR signaling could reverse mitochondrial dysfunction [140]. Despite additional investigations will be needed, compounds that modulate or targeting either these pathways disturbing the mitochondrial network (such as Rapamycin, 5-aminoimidazole-4-carboxamide riboside and resveratrol as or other factors) may represent potential therapeutic approaches to improve and/or prevent the effects of altered mitochondrial function in several diseases including COVID-19.

## 7. Concluding Remarks

COVID-19 continues to be a major threat to the health of the world’s population, and although vaccination has started worldwide, only a small percentage of people have been vaccinated. Therefore, knowing more about the pathogenesis and molecular mechanisms involved in this viral infection will allow for the detection of alternative ways to address this virus not only in treatment, but also in the evaluation of its prognosis and severity in infected patients.

Mitochondria, essential organelles for eukaryotic cells, have proven to be a crucial element in the maintenance, activation, and proliferation of immune cells, providing the metabolic pathways necessary to adapt their dynamics and provide enough energy for their correct function. Furthermore, the presence of MAVS and other proteins in their external structure, and their intimal relation with the immune system, allows for an early and appropriate inflammatory response, with the subsequent elimination of multiple pathogens, mainly viruses. However, an inadequate or exaggerated inflammatory response causes the release of mitochondrial damage products, such as mt-DNA or mt-ROS, into circulation and subsequently activates the immune response and exacerbates the inflammatory state, leading to a worsening of patients’ conditions. As for COVID-19, mitochondria have proven to be an essential component in the replication of SARS-CoV-2 by means of the formation of double-membrane vesicles that propagate its life cycle and inhibit its detection by cellular defense mechanisms. This process causes mitochondrial dysfunction and the subsequent release of mt-DNA, which activates the immune system, activates and releases pro-inflammatory substances, and subsequently generates a cytokine storm. These processes lead to a pro-inflammatory state, which then exacerbates the severity of the disease. While the relevance of mitochondrial dysfunction is essential in the generation of multi-organ failure associated with COVID-19, its relationship with this disease continues to be studied. While more information is needed about the mt-DNA behavior in the COVID-19 inflammatory state, our literature research unravels information that could potentially be helpful in the use of circulating mt-DNA as a potential biomarker for evaluating the prognosis and severity of patients with COVID-19 in evaluating mitochondrial dysfunction, thus allowing for the classification of patients among those who need ICU management or outpatient care and the prediction of the need for future hospitalization. 

## Figures and Tables

**Table 1:** Mechanisms of mitochondrial damage in multiple viral infections.

Virus (Family)	Aim and Study Design	Pathological Mechanism Associated with Mitochondria	Ref
Zika (Flaviviridae)	This study analyzed the consequences of ZIKV infection in iPSC-derived astrocytes present in mice and post-mortem infected neonate brains.	The ZKV replicate in the ER. Ca^2+^ released from the ER to the cytoplasm was taken up by the mitochondria, leading to an increase in ROS production and mitochondrial dysfunction.	[15]
Hepatitis C (Flaviviridae)	This review summarizes the mechanism involved in ROS formation in HCV-replicating cells and describes the intervention of HCV with ROS-detoxifying systems.	HCV replication induced ER stress, which released Ca^2+^ into the cytoplasm, causing an increased Ca^2+^ influx into the mitochondria via VDACs, thus inducing ROS production.	[81]
Japanese encephalitis (Flaviviridae)	Compound CW-33, a synthesized intermediate fluoroquinolone derivative, was investigated to evaluate its antiviral activities against JEV in baby hamster kidneys and human medulloblastoma.	JEV triggered a Ca^2+^ overload, causing a mitochondrial membrane potential alteration.	[83]
Dengue (Flaviviridae)	Freshly isolated platelets from patients infected with dengue were evaluated for the activation of markers and cell death pathways and mitochondrial alterations.	Platelets from dengue-infected patients exhibited classic signs of apoptotic intrinsic pathways that included an increased mitochondrial depolarization, surface phosphatidylserine exposure, and activation of caspase-3 and 9. Mitochondrial dysfunction was followed by the activation of apoptosis in platelets, contributing to thrombocytopenia.	[85]
Dengue (Flaviviridae)	To explore if DENV can affect mitochondrial function and dynamics, such as fusion and fission, a mitochondrion intermixing experiment in two stable A549 cell lines infected with DENV was performed.	The mitochondrial fusion was impaired in dengue virus-infected cells because of the cleaving of two mitofusins (Mfn1-Mfn2) by the dengue virus protease, NS2B3. This led to an increased virus replication and disruption of the mitochondrial membrane potential.	[86]
Dengue virus (Flaviviridae)	Evaluated the functions of NS4B, a DENV non-structural protein associated with a promising drug target for this virus (in relation to the alteration of mitochondrial proteins and morphology), the NS4B in DENV-infected cells were elucidated.	Dengue’s protein, NS4B, induced mitochondria elongation, compromising the integrity of MAM, which are sites of the ER-mitochondria interface critical for innate immune signaling. This promotes virus replication and the activation of interferon responses.	[87]
Hepatitis C Virus (Flaviviridae)	In this study, the ability of HCV NS3-4A protease to cleave peroxisomal and mitochondrial MAVS was analyzed, as well as whether this would have any effect on the cellular antiviral response, by creating a Myc-tagged mutant of MAVS.	The HCV NS3-4A protease cleaved the mitochondrial MAVS, inhibiting the downstream response by blocking the RIG-I-like (RLR) signaling from peroxisomes, thus inhibiting the antiviral gene expression.	[82]
Dengue virus (Flaviviridae)	This paper investigated the role of human dendritic cells in the dengue virus infection.	The dendritic cells infected by the dengue virus showed hypertrophy and proliferation of the ER, as well as swollen mitochondria.	[88]
Dengue virus (Flaviviridae)	Exanimating bone marrow-derived dendritic cells prepared from Tlr9-knockout mice, a previously unrecognized phenomenon in which DENV infection activates TLR9 signaling (important sensors that recognize pathogen-associated molecular patterns) was unraveled by inducing mt-DNA release.	Dengue virus infection induced an mt-DNA release into the cytosol, activating the TLR9 signal pathway and leading to the production of IFNs. This caused ROS generation and inflammasome activation.	[84]
West Nile (Flaviviridae)	In this in vitro study, cell death in the brain-derived tumor cell line, T98G, was induced to illuminate the molecular mechanism of WNV-induced neural cell death.	WNV replication decreased cell viability and induced apoptosis. The intrinsic apoptosis pathway established the signaling of pro-apoptotic proteins such as Bax, which trigger the mitochondrion OM, followed by cytochrome C release from the mitochondria into the cytoplasm.	[89]
Dengue (Flaviviridae)	To confirm the interaction between GR978, a cellular chaperone, and VDAC and DENV E protein, HEK293T/17 cells infected with DENV were used to determine the percentage of infection by flow cytometry.	An interaction between DENV proteins, GRP78 and VDAC, located in the mitochondrial OMM, resulted in the movement of VDAC towards the ER, causing the formation of pores in the mitochondrial membrane and the subsequent release of cytochrome C in the cytosol.	[90]
Japanese Encephalitis (Flaviviridae)	To address the role of fatty-acid β-oxidation in JEV infection, the oxygen consumption rate in JEV-infected cells cultured with or without LCFA palmitate was measured.	JEV nonstructural protein 5 (NS5) interacted with the mitochondrial trifunctional protein, an enzyme complex involved in LCFA B-oxidation, enhancing cytokine production and contributing to JEV pathogenesis.	[91]
Dengue (Flaviviridae)	In this study, the associations between dengue virus-induced cell death and mitochondrial function in HepG2, a human hepatoma cell line, were evaluated.	HepG2 cells infected with the dengue virus suffer continuous metabolic stress, promoting mitochondrial bioenergetics changes, such as a cellular respiration increase, causing mitochondrial swelling and other morphological changes and inducing mitochondrial dysfunction and alterations in the cellular ATP balance.	[92]
Respiratory syncytial (Paramyxoviridae)	Employing bioenergetic measurements, mitochondrial redox-sensitive dye, and high-resolution quantitative imaging, the importance of mitochondrial complex I in RSV infection was studied.	RSV attacked the mitochondrial Complex I subunit knock-out (KO) cells, leading to a mitochondrial respiration decrease and an increased ROS, which favors RSV infection.	[93]
Hepatitis B (Hepadnaviridae)	In this review, the interaction between mitochondria and oncoviruses, particularly in hepatitis B and C and human papilloma and HIV viruses, were discussed.	HBx, a protein of the HBV, targets and integrates into the mitochondrial OMM, inducing ROS overproduction and mt-DNA oxidative damage.	[80]
Hepatitis C (Flaviviridae)		The HCV core protein disturbs both the ER and the mitochondrial OMM, inducing a specific inhibition of Complex I, which adapts the mitochondria for hypoxia and enhances the ROS production at the same time. Meanwhile, the virus core protein facilitates ER Ca^2+^ release and increases the mitochondrial Ca^2+^ uptake, thus increasing the ROS production.	
Papillomavirus (Papillomaviridae)		HPV 18 E2 interacted directly with the mitochondrial respiratory chain and increased the mitochondrial ROS release.	
Human Immunodeficiency (Retroviridae)		HIV targets Mfn2 and reduces its protein level, damaging the integrity of the outer mitochondrial membrane and inducing a progressive mitochondrial deformation.	

Abbreviations: Ref: reference. ATP: adenosine triphosphate. DENV: dengue virus. ER: endoplasmic reticulum. HCV: hepatitis C virus. HIV: human immunodeficiency virus. HPV: human papillomavirus. JEV: Japanese encephalitis virus. LCFA: long-chain fatty acid. MAM: mitochondria-associated membrane. MAVS: mitochondrial antiviral signaling protein. Mfn: mitofusin. OMM: outer membrane. ROS: reactive oxygen species. VDAC: voltage-dependent anion channel. RSV: respiratory syncytial virus. Ref: reference. RT-PCR: real-time polymerase chain reaction. WNV: West Nile virus. ZIKV: Zika virus.
